# Spiral breast computed tomography (CT): signal-to-noise and dose optimization using 3D-printed phantoms

**DOI:** 10.1007/s00330-020-07549-3

**Published:** 2020-12-02

**Authors:** Manon Germann, Sojin Shim, Florian Angst, Natalia Saltybaeva, Andreas Boss

**Affiliations:** Institute of Diagnostic and Interventional Radiology, University Hospital Zurich, Rämistr. 100, 8091 Zurich, Switzerland

**Keywords:** Spiral CT, Breast cancer, Monte Carlo method, Radiation dosage, Signal-to-noise ratio

## Abstract

**Objectives:**

To investigate the dependence of signal-to-noise ratio (SNR) and calculated average dose per volume of spiral breast-CT (B-CT) on breast size and breast density and to provide a guideline for choosing the optimal tube current for each B-CT examination.

**Materials and methods:**

Three representative B-CT datasets (small, medium, large breast size) were chosen to create 3D-printed breast phantoms. The phantoms were filled with four different agarose-oil-emulsions mimicking differences in breast densities. Phantoms were scanned in a B-CT system with systematic variation of the tube current (6, 12.5, 25, 32, 40, 50, 64, 80, 100, 125 mA). Evaluation of SNR and the average dose per volume using Monte Carlo simulations were performed for high (HR) and standard (STD) spatial resolution.

**Results:**

SNR and average dose per volume increased with increasing tube current. Artifacts had negligible influence on image evaluation. SNR values ≥ 35 (HR) and ≥ 100 (STD) offer sufficient image quality for clinical evaluation with SNR being more dependent on breast density than on breast size. For an average absorbed dose limit of 6.5 mGy for the medium and large phantoms and 7 mGy for the small phantom, optimal tube currents were either 25 or 32 mA.

**Conclusions:**

B-CT offers the possibility to vary the X-ray tube current, allowing image quality optimization based on individual patient’s characteristics such as breast size and density. This study describes the optimal B-CT acquisition parameters, which provide diagnostic image quality for various breast sizes and densities, while keeping the average dose at a level similar to digital mammography.

**Key Points:**

*• Image quality optimization based on breast size and density varying the tube current using spiral B-CT.*

**Supplementary Information:**

The online version contains supplementary material available at 10.1007/s00330-020-07549-3.

## Introduction

Worldwide, breast cancer (BC) is not only the most commonly diagnosed cancer but also the leading cause of cancer death among women. In most countries in transition, the incidence rates of BC have increased during the last decades. The incidence of BC in Europe is among the world’s highest, which is why the prevention of BC is a major public health concern in European countries [1].

Breast cancer screening in asymptomatic women has been shown to reduce BC-related mortality [2, 3]. Although there are still some controversies about the overall benefit of BC screening programs, they exist in most European countries [4]. Digital two-view mammography (DM) is the current standard technique for screening and diagnosis of BC. Besides the necessity of painful breast compression, a major issue of DM is the relatively high frequency of false-positive test results, thus a low specificity. Due to tissue overlapping, the sensitivity of DM is reduced in dense breasts, which might lead to small tumors being missed [5, 6]. In addition, breast implants impede the examination of the breast using DM.

Another possible screening technique is digital breast tomosynthesis (DBT) which involves acquiring several low-dose two-dimensional projections by different X-ray tube angles that are used to reconstruct a specific number of image slices comparable to conventional tomography. DBT can be conducted instead or in addition to DM. Reducing the tissue overlapping effect, its application improves sensitivity and specificity significantly [7, 8]. DBT offers very high in-plane resolution but due to a limited rotation angle, only an incomplete set of projections is obtained resulting in a suboptimal *z*-axis resolution [9]. Similar to DM, DBT requires breast compression.

More recently, dedicated spiral breast-CT (B-CT) systems have been introduced and have proved to be a potential alternative to DM and DBT. Due to true 3D image acquisition, B-CT resolution is not only better but also isotropic compared to DM and DBT without painful breast compression. The use of photon-counting detector technology offers a resolution of better than 100 μm for both in-plane and z-resolution. The technology allows B-CT to have more efficient dose utilization and thus reduced radiation dose to levels similar to mammography and tomosynthesis [10–12]. Initial experience with the clinical use of a new dedicated B-CT with photon-counting detector has shown that it can provide high-quality images which are of particular value for BC screening and diagnosis.

Unlike other CT systems, the B-CT requires all scan parameters to be set before acquiring the datasets. However, detailed instructions about the choice of the optimal tube current depending on breast size and density in correlation to the desired signal-to-noise ratio (SNR) and applied dose were not yet addressed.

The purpose of our study is to systematically evaluate the influence of breast density, breast size, and X-ray tube current on SNR and dose levels. Moreover, we aim at providing a table showing the optimal tube current depending on the individual breast size and density, which provides optimal image quality while maintaining sufficient control of the applied average dose.

## Materials and methods

### Datasets

The retrospective evaluation of the datasets was approved by the local ethics committee. Patients gave written informed consent to the retrospective analysis of their data. The image datasets for 3D-printing were acquired in women referred for breast cancer screening without pathological findings. The scans were taken in a prone position using a dedicated spiral B-CT system (nu:view, AB-CT – Advanced Breast-CT GmbH). Three representative CT datasets from patients with different breast sizes (small, medium, large) were chosen to create 3D printable surface models.

### 3D printed breast models

**Fig. 1:**
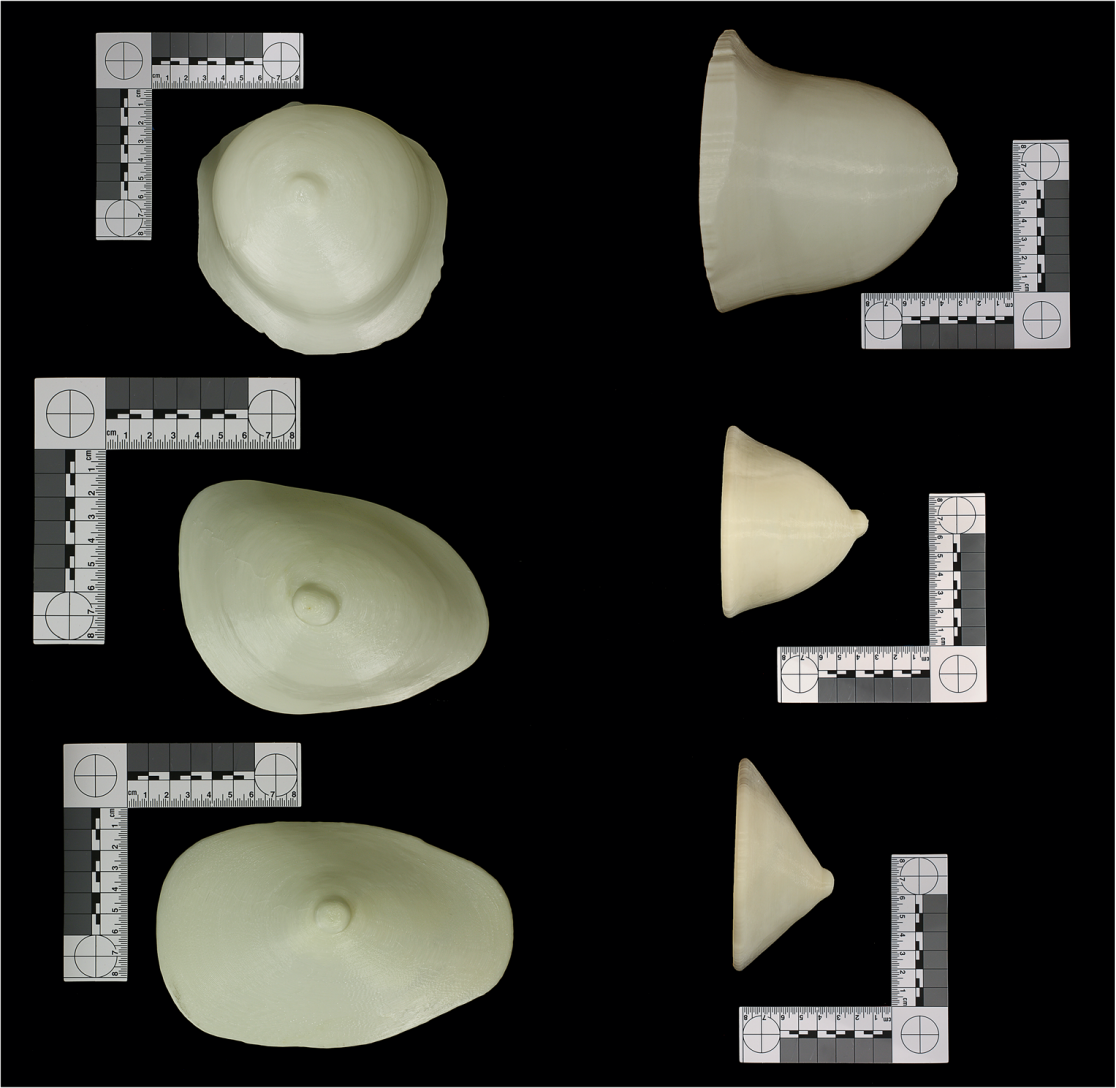
3D printed breast phantoms, ap and lateral view

To create a 3D printable hollow breast model, the breast image files in digital imaging and communications medicine (DICOM) format of the CT datasets were transferred to 3D reconstruction software (Mimics inPrint 3.0, Materialise). The surface models of the three breasts were printed by a 3D printer (Stratasys F370, alphacam swiss GmbH). The machine used ABS Ivory, a high-strength material, for 3D-printing. To hold structures in place, the 3D printer used support material which was washed away by immersing the models into a lye bath after being printed. The measured filling volumes for the different breasts were small (248 ml), medium (358 ml), and large (1067 ml). Figure 1 shows the 3D printed hollow models.

### Tissue phantom (filling)

To imitate different breast densities, emulsions of a 1.5% agar solution (Bacto Agar, Becton Dickinson) and generally available plant oil (containing rapeseed oil and soy oil) were generated. Thereby, the agar solution represented the fibroglandular breast tissue whereas plant oil represented fatty tissue. In total, 30 g of soy lecithin (DAS gesunde PLUS, dm-drogerie markt GmbH + Co) dissolved in 250 ml of water was used as an emulsifier.

Four emulsions of 1 l each were generated representing the four different types of the American College of Radiology (ACR) classification (4^th^ edition) which categorizes the breast density based on the estimated percentage of fibroglandular tissue compared to the whole breast as follows: 1, < 25% glandular density; 2, 25–50% glandular density; 3, 51–75% glandular density; and 4, > 75% glandular density. For our mixtures, we chose the mean value of each category respectively: 1, 12.5% Agar; 2, 37.5% Agar; 3, 62.5% Agar; 4, 87.5% Agar. The exact amounts of the agar and oil are described in Table 1.

**Table 1:** Composition of oil agar emulsions

Components	Mixture 1, 87.5% fat	Mixture 2, 62.5% fat	Mixture, 3 37.5% fat	Mixture 4, 12.5% fat
1.5% agar solution	119 ml	356 ml	594 ml	831 ml
Plant oil	831 ml	594 ml	356 ml	119 ml
Lecithin solution	50 ml	50 ml	50 ml	50 ml
Total	1 l	1 l	1 l	1 l

### Phantom scans

Examinations were performed placing the phantoms in a dedicated spiral B-CT system (nu:view, AB-CT – Advanced Breast-CT GmbH). The phantoms were hung up on a holding device to ensure their complete display on the CT-images. Appendix 6 shows an example of a CT image of high and low breast tissue density in two different patients who signed informed consent for the scientific evaluation of the data in a prospective study, which was approved by the local ethics committee.

The spiral B-CT system is equipped with a photon-counting detector with a cadmium telluride (CdTe) sensor resulting in a detector pixel size of (0.1 mm)^2^ and a total detector area of 280 × 50 mm^2^. The maximum field of measurement has a diameter of 200 mm and a length of 160 mm whereby the scanned length can be selected from three levels (80, 120, or 160 mm). The acquisition time varies according to the chosen field length (7–12 s) and is adjusted automatically, depending on the scan length.

To achieve sufficient contrast for the visibility of microcalcifications, the X-ray tube voltage is fixed at 60 kV. The tube current can be selected from 10 values between 5 and 125 mA (6, 12.5, 25, 32, 40, 50, 64, 80, 100, or 125 mA). More acquisition parameters of the B-CT are reported in Table 2. Two modes exist for data acquisition: the “HighRes (HR)” acquisition mode achieves higher spatial resolution than the second “Standard (STD)” acquisition mode, however thereby reducing SNR (due to the smaller voxel size) [12]. Each of the three phantoms consequently filled with the four different mixtures was scanned in HR mode at all 10 possible tube current values resulting in a total of 120 scans.

**Table 2:** B-CT parameters

X-ray tube current	5–125 mA
X-ray tube voltage	60 kV
Data acquisition rate	Up to 1000 Hz
Number of projections	Up to 2000 per 360°
Number of reconstruction images*	STD 313–589; HR 626–1178
Reconstruction field of measurement	200 × 80–160 mm
Acquisition time*	7 s (small, medium), 12 s (large)

*STD*, standard reconstruction; *HR*, high-resolution reconstruction

*Depending on the scan length

### Evaluation

Image analysis was performed on a PACS clinical workstation using radiographic imaging display software (AGFA Impax 6). DICOM image data were imported to PACS and reconstructed to 0.15-mm coronal slices (high-resolution reconstruction, HR) and 0.3-mm coronal slices (standard reconstruction, STD) using a filtered back-projection reconstruction algorithm. Qualitative image quality was assessed regarding complete depiction of the phantom, and presence of imaging artifacts or air bubbles in the agar-oil emulsion, which was done by one single reader.

Quantitative image quality was evaluated by computation of signal-to-noise ratios (SNR). Thereby, the average Hounsfield unit (HU) of the homogeneous mixture (μ_mix_) was measured placing “regions of interest” (ROIs) of different sizes (> 1000 mm^2^ for the large, > 500 mm^2^ for the medium, and > 200 mm^2^ for the small breast) in areas with minimal air bubbles avoiding imaging artifacts. The average HU in the background was measured in the same way (μ_air_, ROI size 150–200 mm^2^). ROIs were placed to the same locations on the images reconstructed applying HR or STD reconstruction modes. To obtain the SNR, the signal difference between the homogeneous mixture and the surrounding air was divided by the standard deviation of the background (σ_air_) for both reconstruction methods using Eq. 1. The standard deviation of the SNR was calculated using error propagation according to Eq. 2 measuring in the datasets of the tube current of 25, 32, and 40 mA on three different slices; all other SNR values were calculated with single SNR measurements.

1 $$ SNR=\frac{\mu_{\mathrm{mixture}}-{\mu}_{\mathrm{air}}}{\sigma_{\mathrm{air}}} $$

2 $$ {\sigma}_{SNR}=\frac{\sqrt{\sigma_{\mathrm{mixture}}^2+{\sigma}_{\mathrm{air}}^2}}{\sigma_{\mathrm{air}}} $$

### Evaluation of average dose by MC simulation

3D dose distributions were obtained using a commercially available MC simulation (AB-CT – Advanced Breast-CT GmbH) based on ImpactMC [13–15]. The phantom was segmented from the surrounding air based on a thresholding method and considered as a homogeneous breast tissue material in the corresponding breast compositions.

The following scan parameters were applied in the MC simulation: spiral mode, 2-s rotation time, 2000 projections per rotation, 31.53-mm collimation length, and a pitch of 1.05. Total acquisition time depends on the object’s length and equals to 7 s, 9.5 s, and 12 s for 8-, 12-, and 16-cm scan length, respectively. The tube voltage was set at 60 kVp and the tube current was varied at 6, 12.5, 25, 32, 40, 50, 64, 80, 100, and 125 mA. The X-ray tube’s anode angle was 10°, and a beryllium and aluminum filter was applied.

1.0E6 photon histories were calculated for each MC simulation. The average dose in the breast phantom’s volume was acquired from the dose distribution calculated by the MC simulation. The dose calculation was conducted on the 3D breast image reconstructed in STD mode.

### Validation of the Monte Carlo simulation by dose measurements using meta-oxide-semiconductor field-effect transistor sensors

The dose measurements were performed by meta-oxide-semiconductor field-effect transistor (MOSFET) sensors (BMC MOSFETs TN-502RD-H, Best Medical) on the 3D printed breast phantom. The MOSFET sensors measured the local surface dose of the phantom during the scans in the HR acquisition mode. 3D dose distributions over the phantoms were obtained using the MC simulation. The measured and simulated doses were then compared to validate the MC simulation.

## Results

### Subjective image quality evaluation

Regularly, ring artifacts were present in the scans taken with tube currents 25 mA and above, becoming stronger with increasing tube current values. However, in all scans, a significant influence of the ring artifacts on the quantitative evaluation could be avoided due to manual ROI placement between rings. Air bubbles were present in all the scans but areas with only minimal air were available for all measurements.

### Objective image quality evaluation

**Fig. 2:**
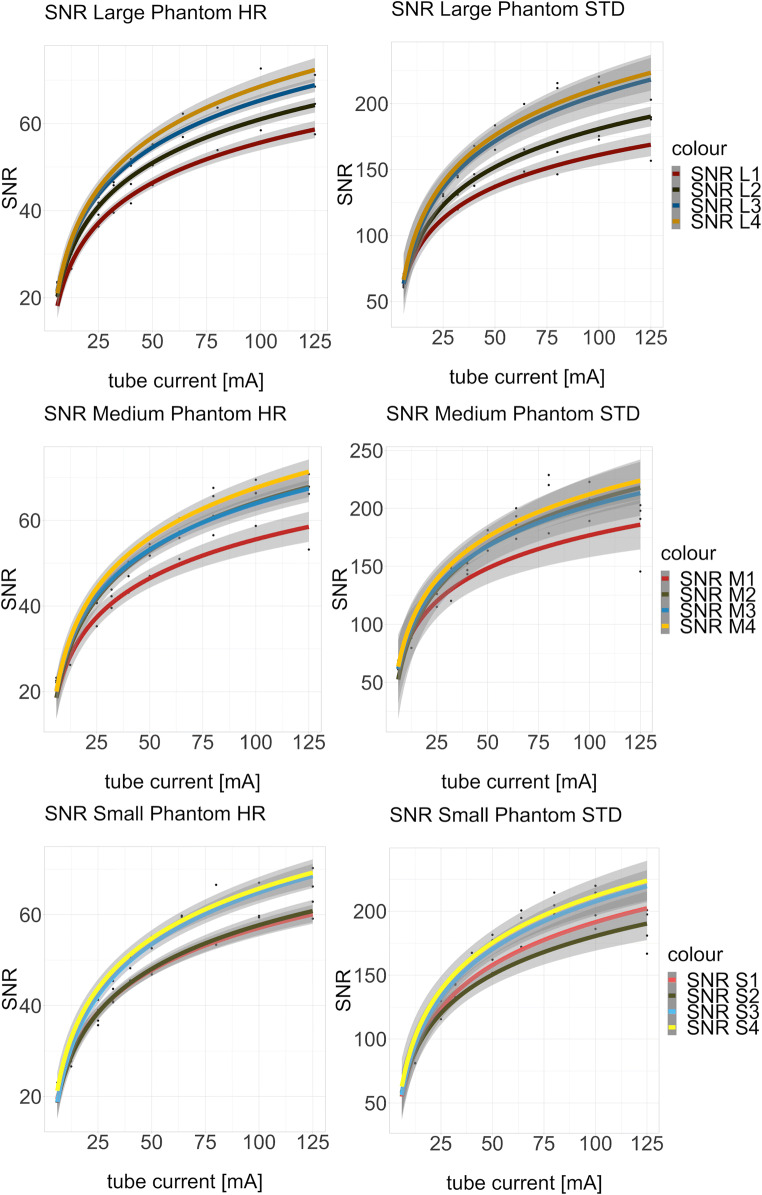
Logistic regression curves of SNR for different breast sizes and compositions

**Fig. 3:**
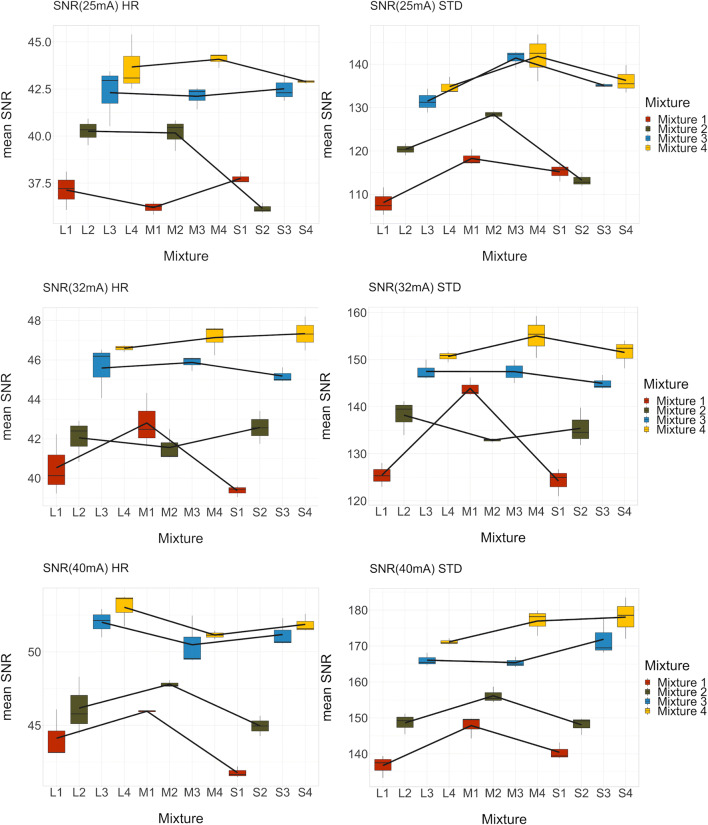
Bar plots displaying mean SNR for different breast sizes and densities

**Fig. 4:**
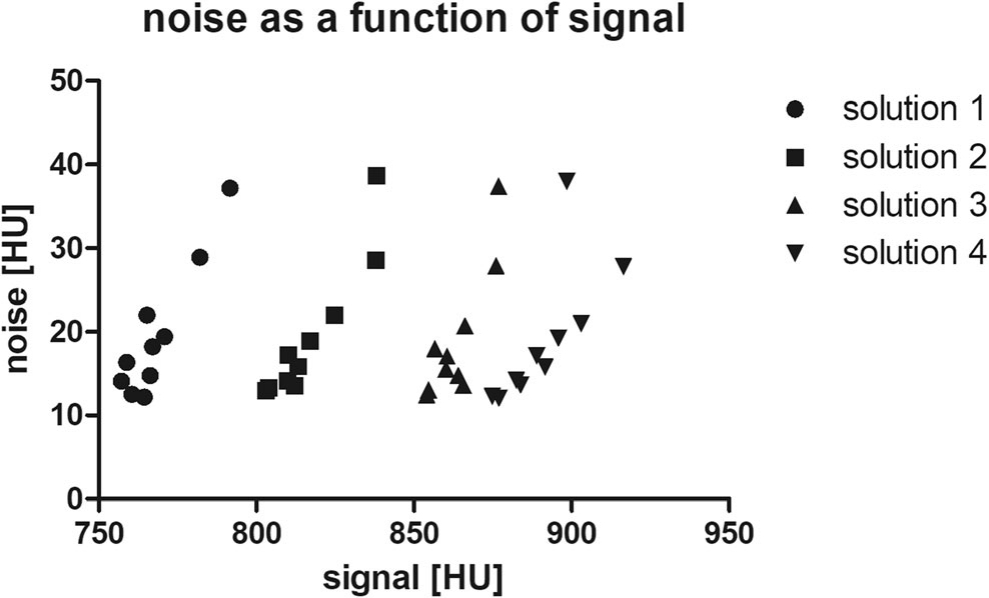
Plot shows the noise as a function of the signal intensity for the large phantom and all 4 solutions. For each solution, 10 measurement points are depicted corresponding to the 10 different tube currents. The highest noise value for each solution corresponds to the lowest tube current. It can be seen that both noise and signal increase with decreasing tube current

SNR of the phantoms at the different tube current values of HR and STD reconstructions are reported in Appendices 3, 4, and 5. Overall SNR values were higher in STD reconstructions than in HR reconstructions. A logarithmic regression was applied to describe the correlation between tube current and SNR as depicted in Fig. 2. In the range from 25 to 50 mA, a pseudolinear relation between the tube current and the SNR was identified, whereas, with tube currents larger than 64 mA, the SNR increase showed a saturation behavior. By increasing the tube current to levels above 64 mA, no substantial increase of the image quality could be achieved. Figure 3 shows the diagrams for the three clinically most important tube current settings (25, 32, and 40 mA) from top to bottom, high-resolution (HR), and standard-resolution (STD) on the right side. SNR increases with increasing density of the mixture, whereas no clear correlation between SNR and phantom size can be identified. An example plot of the noise as a function of the mean signal value is provided in Fig. 4.

### Validation of the Monte Carlo calculations: surface dose comparison with MOSFET

The local surface doses obtained from 3D dose calculations of the MC program exhibited an acceptable difference to the surface doses measured by the MOSFETs (see Table 3). The difference between the measured and the calculated dose was less than 23% for each measurement.

**Table 3:** Comparison of measured and calculated surface doses for validation of the MC simulation

	MOSFET measurement	MC simulation	Difference
	(mGy)	(mGy)	(%)
Large	10.0 ± 0.77	8.6 ± 1.01	- 13.7 ± 6.26
Medium	6.8 ± 0.35	8.0 ± 0.80	18.1 ± 8.41
Small	7.2 ± 0.50	6.7 ± 0.37	- 7.7 ± 1.40

### Dose distribution

Appendix 7 shows a typical example of a dose distribution in the different phantoms calculated by MC simulation. Due to spiral CT scanning, the dose distribution of each transactional slide shows an asymmetric dose distribution. The peripheries are more irradiated than the center due to the high X-ray attenuation at the peripheries. As the X-ray beam travels a greater distance in the large compared to the small breast and, therefore, is more attenuated, the dose fall-off is greater in the large breast phantom, which leads to lower dose at the center for the large phantom compared to the smaller phantom. The effect of the dose attenuation at the periphery is demonstrated in Appendix 2 where line plots of the transactional absorbed dose distributions calculated in the MC simulation are presented.

### Average absorbed dose as a function of breast size and glandularity

**Fig. 5:**
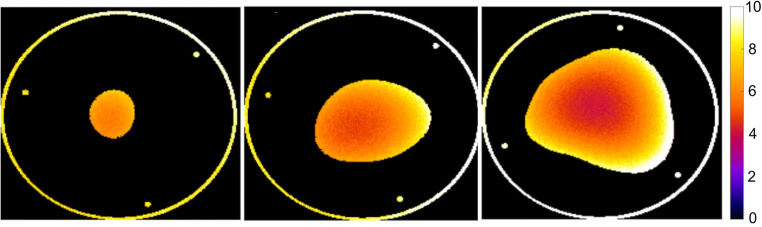
Average absorbed dose in mGy as a function of the X-ray tube current

The average dose over the breast is smaller for the large breast than the small breast as a result of the dose distribution. The average absorbed doses calculated by MC simulation are presented in Fig. 5.

**Fig. 6:**
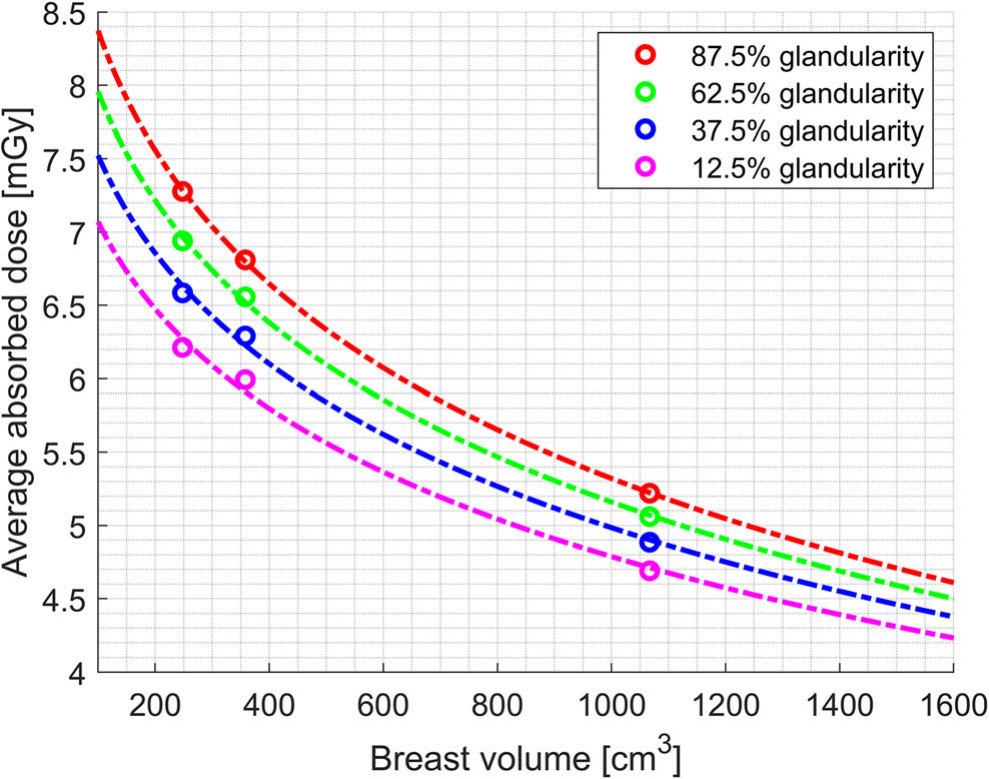
Average absorbed dose as a function of breast volume and density at 25-mA X-ray tube current

The simulated average absorbed dose over the breast as a function of breast volume and glandularity at 25 mA of X-ray tube current is demonstrated in Fig. 6. The average absorbed dose exhibits an exponential behavior depending on the breast size and a linear correlation to the glandularity of the breast (*p* < 0.05). The resulting values of the average absorbed dose from the simulation and regression at 25 mA of X-ray tube current are presented in Appendix 1. Reflecting the linear correlation of the dose to the X-ray tube current, the average absorbed dose equation is acquired as a function of the dose factor derived for each breast volume and glandularity of 3D phantoms and the mixture fillings in Eq. 3. *F*(*b*reast volume, glandularity) denotes the dose factor as a function of breast volume and glandularity, and it denotes the X-ray tube current. The lookup table of the factors is presented in Table 4.

3 $$ \mathrm{Average}\ \mathrm{absorbed}\ \mathrm{dose}=F\left(\mathrm{breast}\ \mathrm{volume},\mathrm{glandularity}\right)\times I $$

**Table 4:** Lookup table of radiation dose factor as a function of breast volume and glandularity

***F*(breast volume, glandularity) (mGy/mA)**		**Breast volume (cm**^**3**^**)**		
		**248**	**358**	**1067**
**Glandularity (%)**	**12.5**	0.25	0.24	0.19
	**37.8**	0.27	0.25	0.20
	**62.5**	0.28	0.26	0.20
	**87.5**	0.29	0.27	0.21

### Optimization of acquisition parameters regarding image quality and dose

SNR values ≥ 35 (HR) and ≥ 100 (STD) offer sufficient image quality for clinical evaluation and were set as minimum values. Table 5 shows which tube current should be selected to achieve a reasonable image quality at the dose below 6.5 mGy. In order to get sufficient image quality, the dose limit for the small breast phantom had to be increased to 7 mGy.

**Table 5:** Optimal tube current for conditions: SNR ≥ 35 (HR), SNR ≥ 100 (STD), dose ≤ 6.5 mGy, and tube current ≥ 25 mA

	Mixture 1	Mixture 2	Mixture 3	Mixture 4
Large	25 mA (5.22 mGy)	32 mA (6.48 mGy)	32 mA (6.25 mGy)	32 mA (6 mGy)
Medium	25 mA (6.81 mGy)*	25 mA (6.56 mGy)*	25 mA (6.29 mGy)	25 mA (5.99 mGy)
Small	25 mA (7.28 mGy)*	25 mA (6.94 mGy)*	25 mA (6.58 mGy)*	25 mA (6.21 mGy)

*> 6.5 mGy

## Discussion

The average absorbed dose of breasts for the optimal image quality decreases with decreasing breast size for the same breast density and increases with increasing breast density. SNR increases with breast density; however, no systematic dependence of SNR on breast size for the same X-ray tube current was found. Individual adjustment of the tube current according to patient’s characteristics allows to obtain images of suitable quality while keeping sufficient control of the applied dose levels.

Compared to conventional mammography systems, scan parameters in spiral B-CT need to be manually chosen before the examination is performed. It is known that both absorbed dose and image quality depend on individual patient characteristics such as breast size and density. Therefore, it is important to know in advance which tube current results in an image stack of optimum quality at the lowest possible dose depending on the previously mentioned patient characteristics. From our recommendation table for B-CT examinations, it can be seen that dose levels can be kept between 5.2 and 7.3 mGy for all breast densities and breast size with the highest dose levels obtained in small breasts with low density.

In cone-beam B-CT, usually two orthogonal low-dose scout images of the breast are obtained before image acquisition to set the optimal tube current. To keep the radiation dose as low as possible, no scout images are acquired in the spiral B-CT; therefore, the tube current has to be set before image acquisition. Both cone-beam and spiral B-CT offer the possibility to acquire true 3D datasets. In contrast to cone-beam B-CT, which achieves a lower spatial resolution than DM, spiral B-CT offers a resolution of better than 100 μm for both in-plane and z-resolution with a better depiction of microcalcifications [10, 16].

Our study has shown that by varying the tube current depending on breast density and size, sufficient image quality can be achieved at dose levels similar to DM. Thereby, the SNR is more dependent on the breast density than on breast volume. The average dose over the breast is higher for the smaller breast than for the larger due to the relatively higher surface area compared to the total volume. The surface area receives higher dose than the inner part of the breast which is relatively less in small breasts.

Meanwhile, most European countries implemented BC screening programs using mammography which resulted in a reduction of breast cancer (BC) mortality [2, 3]. However, tissue overlapping or dense glandular tissue (ACR categories c and d) can mask small tumors and thus reduce the sensitivity of DM. Especially in women with dense breast tissue or breast implants, the accuracy of mammography breast cancer screening is substantially reduced. Moreover, painful breast compression is required for DM, which does not only lead to patient discomfort but could also discourage women from participating in screening programs [5, 6, 17]. The recently introduced spiral B-CT system offers breast image acquisition without painful compression. This might encourage women to take part in BC screening programs in the future thus rendering screening programs more effective.

Several studies have shown very promising results regarding the use of dedicated spiral B-CT in the screening and diagnosis of breast cancer. Several studies have shown B-CT to be a good alternative to conventional mammography in terms of both radiation dose and quality [11, 12, 18, 19]. In principle, dynamic scans after contrast-agent injection are possible. Scanning in STD mode, the dose could keep at an acceptable level even after two or more acquisitions. It has been demonstrated that even low-dose perfusion CT of the breast in a prone position is possible and shows significant differences in breast lesions compared to normal breast tissue [18, 20].

One disadvantage of the current B-CT device is the relatively long reconstruction time of the 3D datasets, which takes approximately 20 min to be completed whereas DM images are available immediately after acquisition. No scout images are available, so that breast density cannot be assessed from the B-CT before the scan is completed. Therefore, previously available mammography is helpful for the choosing of the best tube current setting. B-CT systems are known for limited coverage of the axilla compared to DM. Therefore, patient positioning is extremely important and needs to be done carefully. Additional sonographic evaluation can solve the problem of the axilla extending glandular tissue [16, 19].

Our study has several limitations: Firstly, the data was obtained using 3D printed phantoms filled with homogeneous mixtures. Soft tissue lesions or microcalcifications were not implemented in the phantoms. Therefore, the visibility of soft tissue lesions or microcalcifications could not be assessed. Secondly, validation of the Monte Carlo simulations was conducted by measuring the surface dose of the phantoms using MOSFET sensors; direct dose measurements within the phantoms were not performed. Thirdly, only three different breast sizes were evaluated. However, a larger number of breast phantoms for each breast size would have added little further information. Fourthly, the average dose instead of mean glandular dose was calculated due to the homogeneous mixtures. However, the average dose may be used as a worst-case scenario with mean glandular dose typically being smaller. The reason for that is that the dose in the central parts is typically smaller compared to the dose in the surface region of the breast [12]. Fifthly, we were not able to compute contrast-to-noise ratios in dependence of the tube current or dose, which would have been helpful for choosing the best tube current setting and for comparison to existing literature. This topic will be addressed in a subsequent study requiring new 3D printed phantoms. Sixthly, new reconstruction algorithms such as iterative reconstruction or techniques based on deep learning may be advantageous compared to filtered back-projection. However, the evaluation of these new algorithms was out of the scope of this study.

## Conclusion

In conclusion, adjusting the scan parameters in spiral B-CT based on breast density and breast volume provides optimal image quality at a reasonable dose, individually adapted to the patient characteristics.

## Supplementary information

ESM 1 (DOCX 5911 kb)

## Abbreviations

ACR: American College of Radiology
BC: Breast cancer
B-CT: Breast computed tomography
DBT: Digital breast tomosynthesis
DM: Digital mammography
HR: High-resolution
HU: Hounsfield unit
MC: Monte Carlo
MOSFET: Meta-oxide-semiconductor field-effect transistor
ROI: Region of interest
SNR: Signal-to-noise ratio
STD: Standard resolution
