# Supplementary material for: Spiral breast computed tomography (CT): signal-to-noise and dose optimization using 3D-printed phantoms
Source: Eur Radiol. 2020 Dec 2;31(6):3693–702. doi: 10.1007/s00330-020-07549-3 (PMC8128791; doi:10.1007/s00330-020-07549-3)

**Appendix**

**Appendix 1.** The result of average absorbed dose from the simulation and the regression at X-ray tube current of 25 mA

| **Average absorbed dose [mGy]** | | | **Breast volume [cm^3^]** | | |
| --- | --- | --- | --- | --- | --- |
|  |  |  | **248** | **358** | **1067** |
| **Glandularity [%]** | **12.5** | **Simulation** | 6.21 | 5.99 | 4.69 |
|  |  | **Regression** | 6.27 | 5.91 | 4.71 |
|  | **37.5** | **Simulation** | 6.59 | 6.29 | 4.89 |
|  |  | **Regression** | 6.63 | 6.23 | 4.90 |
|  | **62.5** | **Simulation** | 6.94 | 6.56 | 5.06 |
|  |  | **Regression** | 6.97 | 6.52 | 5.07 |
|  | **87.5** | **Simulation** | 7.28 | 6.81 | 5.22 |
|  |  | **Regression** | 7.28 | 6.80 | 5.22 |

**Appendix 2.** Dose distribution in the three different phantom sizes for the example of mixture 4 scanned with 32mA X-ray tube current.

**
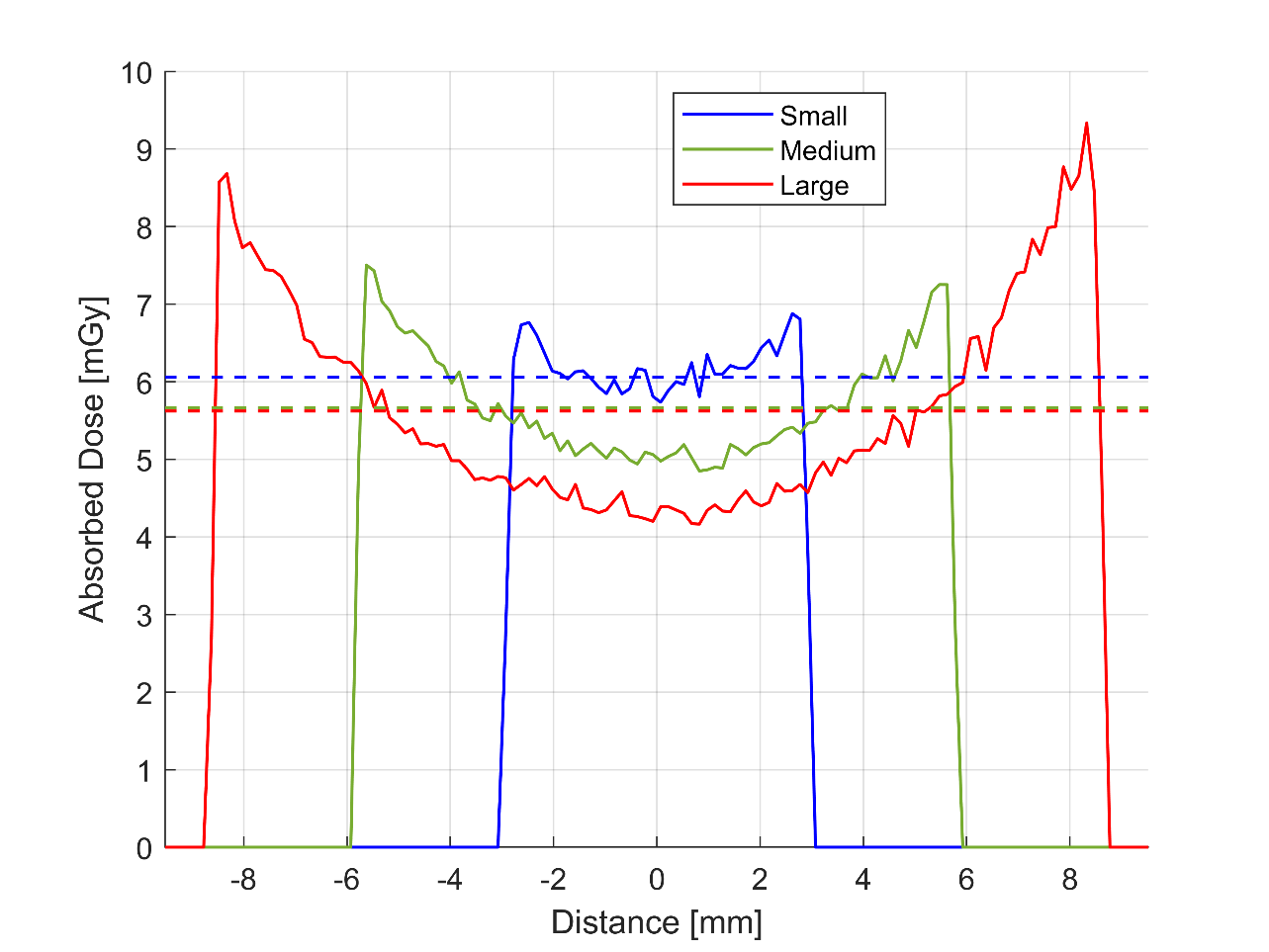
**

**Appendix 3.** Signal-to-noise-ratio (SNR) of High Resolution (HR) Reconstruction depending on the tube current

* L1-4: Large, Mixtures 1-4; M1-4: Medium, Mixtures 1-4; S1-4: Small, Mixtures 1-4

| Tube current [mA] | SNR  L1*  HR | SNR  L2*  HR | SNR  L3*  HR | SNR  L4*  HR | SNR  M1*  HR | SNR  M2*  HR | SNR  M3*  HR | SNR  M4*  HR | SNR  S1*  HR | SNR  S2*  HR | SNR  S3*  HR | SNR  S4*  HR |
| --- | --- | --- | --- | --- | --- | --- | --- | --- | --- | --- | --- | --- |
| 6 | 20.30 | 21.65 | 23.43 | 23.62 | 20.63 | 22.16 | 22.60 | 23.23 | 21.86 | 22.18 | 22.20 | 23.07 |
| 12.5 | 26.63 | 30.01 | 31.42 | 33.03 | 26.24 | 28.72 | 31.16 | 31.68 | 27.75 | 26.61 | 30.36 | 32.54 |
| 25 | 36.35 | 39.07 | 41.83 | 43.08 | 35.28 | 40.67 | 40.77 | 41.61 | 35.69 | 36.69 | 39.05 | 41.27 |
| 32 | 39.53 | 45.83 | 47.59 | 46.54 | 39.55 | 42.29 | 43.84 | 46.30 | 40.88 | 40.71 | 43.68 | 45.41 |
| 40 | 41.68 | 46.12 | 50.32 | 51.83 | 43.13 | 46.97 | 49.18 | 50.33 | 45.51 | 44.66 | 48.23 | 50.61 |
| 50 | 45.86 | 50.43 | 55.25 | 56.57 | 47.02 | 51.78 | 53.62 | 54.45 | 47.42 | 46.86 | 52.60 | 55.15 |
| 64 | 49.92 | 56.92 | 58.46 | 62.28 | 50.99 | 57.42 | 55.92 | 60.47 | 51.44 | 51.67 | 59.53 | 59.79 |
| 80 | 53.90 | 57.65 | 63.65 | 64.70 | 56.55 | 65.67 | 61.06 | 67.61 | 54.76 | 53.39 | 62.45 | 66.56 |
| 100 | 58.46 | 60.79 | 65.39 | 72.65 | 58.71 | 66.43 | 66.33 | 69.50 | 59.40 | 59.73 | 65.17 | 67.02 |
| 125 | 57.54 | 64.53 | 68.48 | 71.18 | 53.20 | 66.20 | 67.88 | 70.85 | 59.13 | 62.85 | 70.25 | 66.20 |

**Appendix 4.** Signal-to-noise-ratio (SNR) of Standard Resolution (STD) Reconstruction depending on the tube current

* L1-4: Large, Mixtures 1-4; M1-4: Medium, Mixtures 1-4; S1-4: Small, Mixtures 1-4

| Tube current  [mA] | SNR  L1*  STD | SNR  L2*  STD | SNR  L3*  STD | SNR  L4*  STD | SNR  M1*  STD | SNR  M2*  STD | SNR  M3*  STD | SNR  M4*  STD | SNR  S1*  STD | SNR  S2*  STD | SNR  S3*  STD | SNR  S4*  STD |
| --- | --- | --- | --- | --- | --- | --- | --- | --- | --- | --- | --- | --- |
| 6 | 60.71 | 61.76 | 62.54 | 71.14 | 61.28 | 63.48 | 66.16 | 68.97 | 57.67 | 58.87 | 61.70 | 66.89 |
| 12.5 | 87.31 | 90.30 | 98.31 | 99.92 | 79.67 | 88.20 | 93.60 | 96.91 | 80.94 | 81.31 | 90.66 | 94.89 |
| 25 | 113.60 | 124.75 | 129.91 | 131.52 | 115.12 | 118.74 | 121.07 | 126.24 | 119.05 | 115.54 | 120.72 | 129.68 |
| 32 | 119.84 | 131.00 | 145.32 | 144.24 | 129.57 | 120.27 | 150.19 | 148.61 | 142.47 | 132.73 | 143.02 | 145.31 |
| 40 | 137.74 | 146.50 | 168.01 | 164.52 | 143.29 | 146.85 | 152.66 | 165.22 | 147.64 | 147.72 | 158.33 | 167.48 |
| 50 | 138.01 | 165.02 | 171.78 | 183.56 | 163.57 | 177.06 | 174.57 | 181.20 | 162.09 | 151.76 | 177.33 | 181.54 |
| 64 | 148.54 | 165.34 | 189.54 | 199.80 | 173.75 | 200.13 | 185.88 | 193.24 | 168.92 | 172.18 | 194.76 | 200.59 |
| 80 | 146.46 | 163.36 | 211.84 | 215.63 | 178.54 | 228.80 | 192.92 | 220.15 | 197.98 | 179.86 | 204.65 | 214.61 |
| 100 | 172.74 | 175.49 | 220.49 | 216.01 | 189.16 | 206.61 | 207.49 | 222.82 | 196.85 | 186.16 | 214.54 | 219.92 |
| 125 | 156.72 | 189.76 | 188.08 | 203.02 | 145.60 | 190.94 | 202.77 | 197.96 | 180.94 | 166.80 | 200.86 | 197.49 |

**Appendix 5.** Signal-to-noise-ratio (SNR) of Standard Resolution (STD) and High Resolution (HR) depending on the tube current

* L1-4: Large, Mixtures 1-4; M1-4: Medium, Mixtures 1-4; S1-4: Small, Mixtures 1-4

| **High Resolution (HR)** | | | | | | | | | | | | |
| --- | --- | --- | --- | --- | --- | --- | --- | --- | --- | --- | --- | --- |
| Tube current [mA] | SNR  L1*  HR | SNR  L2*  HR | SNR  L3*  HR | SNR  L4*  HR | SNR  M1*  HR | SNR  M2*  HR | SNR  M3*  HR | SNR  M4*  HR | SNR  S1*  HR | SNR  S2*  HR | SNR  S3*  HR | SNR  S4*  HR |
| 25 | 37.13±4.96 | 40.26±5.45 | 42.30±5.78 | 43.67±6.37 | 36.20±2.04 | 40.16±2.63 | 42.11±2.69 | 44.08±3.32 | 37.75±1.85 | 36.15±2.29 | 42.51±2.01 | 42.89±2.13 |
| 32 | 40.53±4.44 | 42.05±5.08 | 45.59±5.24 | 46.59±6.32 | 42.80±2.43 | 41.56±2.75 | 45.87±2.75 | 47.14±3.25 | 39.37±1.69 | 42.57±2.32 | 45.18±1.98 | 47.33±2.39 |
| 40 | 44.12±4.49 | 46.17±4.64 | 52.01±5.31 | 53.03±6.29 | 45.96±2.12 | 47.79±2.59 | 50.48±2.64 | 51.14±3.18 | 41.78±1.63 | 44.96±2.58 | 51.18±2.03 | 51.87±2.42 |
| **Standard Resolution (STD)** | | | | | | | | | | | | |
| Tube current [mA] | SNR  L1*  STD | SNR  L2*  STD | SNR  L3*  STD | SNR  L4*  STD | SNR  M1*  STD | SNR  M2*  STD | SNR  M3*  STD | SNR  M4*  STD | SNR  S1*  STD | SNR  S2*  STD | SNR  S3*  STD | SNR  S4*  STD |
| 25 | 108.10±4.25 | 120.37±4.85 | 131.49±6.89 | 134.84±6.23 | 118.28±1.94 | 128.38±2.56 | 141.42±2.79 | 141.81±3.50 | 115.19±1.69 | 113.26±2.71 | 135.13±2.06 | 136.27±2.33 |
| 32 | 125.44±4.15 | 138.20±4.64 | 147.49±5.89 | 150.67±6.19 | 143.89±2.44 | 132.89±2.78 | 147.45±2.85 | 155.01±3.58 | 124.22±1.60 | 135.40±2.04 | 144.96±2.04 | 151.53±2.68 |
| 40 | 136.73±4.22 | 148.61±4.51 | 166.05±5.36 | 171.15±6.19 | 147.86±2.04 | 156.15±2.66 | 165.37±2.94 | 176.95±3.71 | 140.44±1.67 | 148.07±2.25 | 171.87±2.25 | 178.02±2.79 |

**Appendix 6.** Example B-CT images of low breast tissue density (64y old patient; left) and high breast tissue density (55y old patient; right) are displayed.


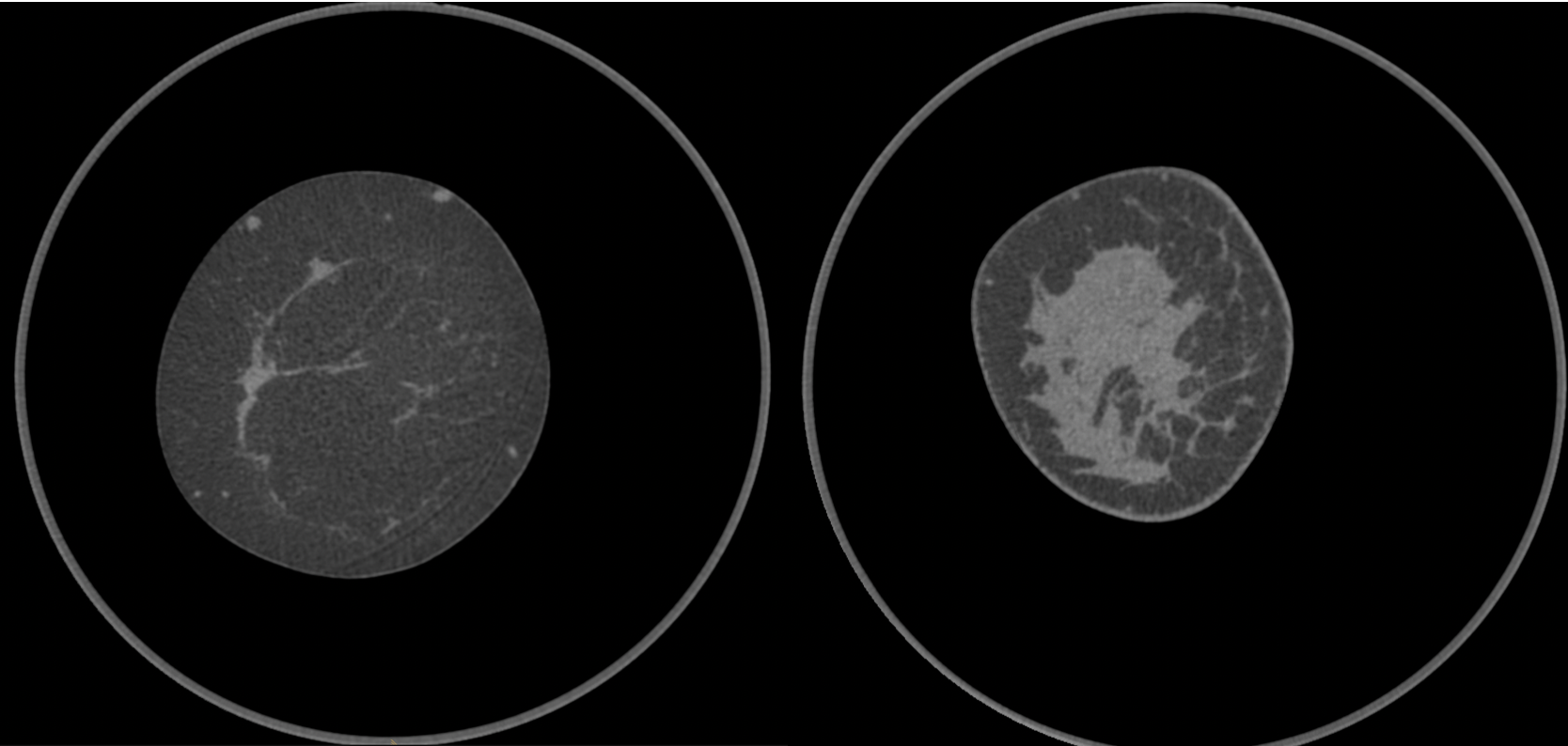


**Appendix 7.** Dose distribution of the phantoms with the material mixture 4 scanned with 32 mA X-ray tube current


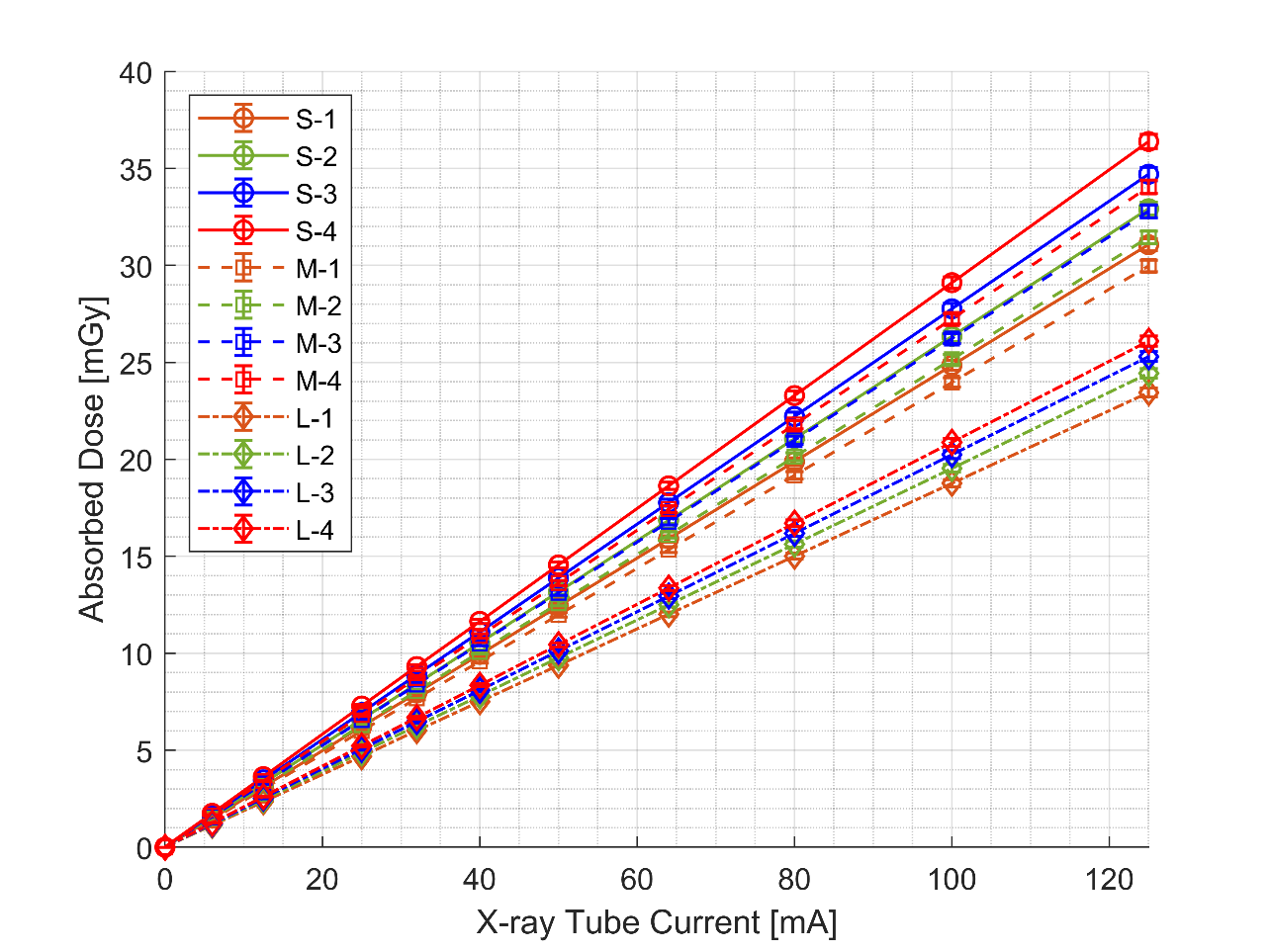

Supplement: Supplementary file 1 — (DOCX 5911 kb) [file 330_2020_7549_MOESM1_ESM.docx]
